# Three-Point Bending Tests of Zirconia Core/Veneer Ceramics for Dental Restorations

**DOI:** 10.1155/2013/831976

**Published:** 2013-02-25

**Authors:** Massimo Marrelli, Carmine Maletta, Francesco Inchingolo, Marco Alfano, Marco Tatullo

**Affiliations:** ^1^Biomedical Section, Tecnologica Research Institute, Via E. Fermi, 88900 Crotone, Italy; ^2^Department of Mechanical Engineering, University of Calabria (UniCAL), 87136 Arcavacata, Cosenza, Italy; ^3^Department of Dental Sciences and Surgery, University of Bari, 70124 Bari, Italy

## Abstract

*Introduction*. The mechanical strength and the surface hardness of commercially available yttrium-doped zirconia were investigated. Furthermore, a comparative study of eight different ceramic veneers, to be used for the production of two-layered all-ceramic restorative systems, was carried out. *Materials and Methods*. Four types of zirconia specimens were analyzed, according to a standard ISO procedure (ISO 6872). Besides, two-layered zirconia-veneer specimens were prepared for three-point bending tests. *Results*. A strong effect of the surface roughness on the mechanical strength of zirconia specimens was observed. Finally, a comparative study of eight commercially available veneering ceramics shows different modes of failure between the selected veneers. *Conclusion*. The results indicate that close attention should be paid to the preparation of zirconia-based crowns and bridges by CAD/CAM process, because surface roughness has an important effect on the mechanical strength of the material. Finally, the results of the mechanical tests on two-layered specimens represent an important support to the choice of the veneering ceramic.

## 1. Introduction

The use of advanced ceramics as restorative dental materials is strongly increasing, owing to the introduction of Computer-Aided Design/Computer-Aided Manufacturing (CAD/CAM) milling techniques which allow the fabrication of large and complex restorations with very high-dimensional accuracy [1, 2]. The most promising production method consists in a soft machining of presintered blocks, which are subsequently sintered at high temperature [3]. As a final step, sintered structures are usually coated using veneering ceramics, in order to obtain two-layered all-ceramic restorative systems with very attractive mechanical properties, good biocompatibility, and excellent esthetic results [4].

Among the ceramic materials for dental applications, the zirconia-based ones are very widespread, because of their transformation toughening capabilities [5, 6]. 

The aim of the present study is to analyze the mechanical behavior of commercially available Y-TZP ceramics for dental applications and to estimate the effects of different processing conditions, which usually occur during production by CAD/CAM techniques. In addition to this, eight commercially available ceramic veneers, to be used for the production of all-ceramic restorations in combination with Y-TZP structures, were analyzed by using three-point bending tests on two-layered specimens. Finally, a systematic comparative analysis of the eight selected ceramic veneers was carried out.

## 2. Materials and Methods

The mechanical strength of a commercial yttria-stabilized zirconia, to be used as core material for the production of crowns and bridges in combination with CAD/CAM techniques, was analyzed by three-point bending tests on standard specimens. Furthermore, the mechanical behavior of eight different types of veneering ceramics was analyzed by flexural tests of two-layered zirconia-veneer specimens and microhardness measurements.

### 2.1. Flexural Tests of Core Specimens

The bending tests of zirconia-based core material were carried out following standard ISO procedures and recommendations (ISO 6872). Beam specimens, with length (*l*) = 25 mm, width (*b*) = 5 mm, and thickness (*t*) = 2 mm, were prepared for flexural tests; all dimensions were measured with an accuracy of ±0.02 mm. The specimens were cut from presintered blocks by using a high-speed cutting machine, provided with a diamond disk (*ϕ*  63 mm), and their size was properly increased in order to take into account the material shrinkage which occurs in the subsequent sintering process.

The three-point bending tests were done under displacement control (cross-head feed rate equal to 1 mm/min) by using a universal testing machine (Instron 8500), with a 5 kN load cell, controlled by a TestStar II (MTS) controller. 

In order to analyze the effect of surface finishing and coloring process on the flexural strength, four different specimen types were produced and tested, for example, Type A, Type B, Type C, and Type D (Table 1).

Type A specimens were tested when sintered. 

Type B specimens were colored before sintering, by using commercial dyes. 

Type C specimens were polished after sintering; in particular, surface polishing was done by using water-cooled carborundum disks, with progressively finer alumina grits, ranging between 400, 800, and 1200, respectively. 

Type D specimens were colored, sintered, and polished. As the surface condition can have a great influence on the mechanical behavior of the core material, the surface roughness of each specimen type was measured by using a contact measuring system (MarSurf III, Mahr). The surface roughness was measured along the longitudinal and transversal direction of the specimens. Five measurements for each direction were carried out, with a traveling distance of 2 mm.

### 2.2. Flexural Tests of Two-Layered Core/Veneer Specimens

Flexural tests of two-layered core/veneer specimens, for each veneering ceramic analyzed, were carried out by using the same equipment and testing parameters described in the previous section. The specimens, with length *l* = 25 mm, width *b* = 5 mm, and total thickness *t* = 2.2 mm, were made by coating 1.1 mm thick zirconia core layers with identical thickness veneering ceramics, by following the manufacturer's directions and instructions. It is worth noting that zirconia substrates were coated and sintered without any preliminary surface treatment.

The specimens were analyzed by three-point bending tests, with a test span equal to 15 mm, and the load was applied on the veneer surface; that is, the veneer layer is subjected to compressive stress. In order to carry out a comparative analysis between the eight selected veneering ceramics, the total strain energy per unit volume was calculated.

In fact, this energetic parameter allows a comparative study between the different types of core/veneer specimens, as it describes the overall mechanical behavior of the two-layered system. The total strain energy per unit volume was calculated from the experimentally measured load-deflection curves, by considering the volume of the specimen between the test spans.

## 3. Results

The results, concerning the experimental tests carried out on zirconia core specimens and on two-layered core/veneer systems, are described in this section. In particular, the results of bending tests performed on zirconia specimens are firstly discussed; then, a comparative analysis of the eight selected ceramic veneers is given. 

### 3.1. Mechanical Strength of Zirconia Core Material

Twenty specimens for each type listed in Table 1 (Types A, B, C, and D) were analyzed, in order to measure the effects of coloring and surface roughness on the mechanical strength; Student's *t*-test at a 95% confidence level was done in order to analyze the difference in strength between the four types of specimens.  Table 2 summarizes the results obtained for surface roughness and flexural strength. As expected, Types C and D (polished specimens) show similar values of surface roughness, as well as Types A and B (nonpolished specimens) (Table 2).

Table 2 clearly shows that surface polishing causes a strong increase in the average values of flexural strength, as well as in Weibull characteristic strength.  Table 2 also shows that the flexural strength of the polished specimens is characterized by smaller standard deviations (and higher Weibull moduli) with respect to the unpolished ones. These results are mainly caused by microscopic surface sharp cracks and scratches, which act as crack initiation sites. These observations are also confirmed by Student's *t*-test which indicates a significant difference in strength between polished and nonpolished specimens, while the coloring process does not significantly affect the mechanical strength.

### 3.2. Mechanical Behavior of Two-Layered Zirconia-Veneer Systems

**Figure 1:**
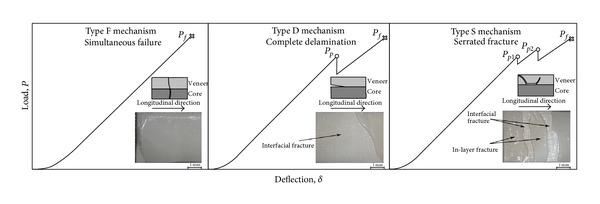
Failure mechanisms of the two-layered zirconia-veneer specimens together with schematic depictions of the force-deflection curves and optical observations of the fracture surfaces.

Ten two-layered specimens for each veneering ceramic were analyzed by three-point bending tests. Three different failure mechanisms have been observed as shown in Figure 1.

- Simultaneous failure of zirconia and veneer (Type F).
- Complete interfacial debonding of the veneer (Type D).
- Serrated fracture of the ceramic veneer (Type S), that is, the crack spreads in the veneer, approaches the zirconia-veneer interface, and then kinks again into the veneer.

In particular, Figure 1 illustrates schematic depictions of the force-deflection curves for the three observed failure mechanisms, and optical observations of the fracture surfaces are also given. Figure 1 shows that a monotonic force-deflection curve is obtained when the simultaneous failure of zirconia and veneer occurs at failure load *P*_*f*_ (Type-F). In this case, the crack, initiated in the zirconia substrate, was only able to extend in the veneer, and interfacial fracture did not occur. It is therefore believed that the material systems, which consistently showed this failure mechanism, were characterized by stronger interactions at the veneer/zirconia interface. On the other hand, nonmonotonic curves with one or multiple intermediate peaks at the load *P*_*pi*_  were also observed and classified as Type D and Type S mechanisms. In both cases, the crack was able to deflect at the zirconia-veneer interface, and therefore interfacial failure did occur. However, while for Type D the deflected crack proceeded all the way along the interface, for Type S it oscillated between the interface and the veneer. The reason for this behavior could be addressed to the relative proportion of interfacial to veneer fracture toughness. Indeed, for a tougher veneer the deflected crack could be entrapped at the interface. However, some of the investigated bimaterial systems have shown multiple failure mechanisms (see Table 3); therefore, it is inferred that process variability may also play a role. Anyway, this point would deserve additional study to be fully understood and therefore it can be certainly considered as a future extension of the present work. 

On the basis of the previous considerations, a first qualitative comparison of the eight selected ceramic veneers was made. In Table 3, the aforementioned failure mechanisms for each type of ceramic veneer are indicated (Table 3).

Table 3 clearly shows that the specimens coated with Sakura Interaction and Ceramco PFZ ceramics resulted in simultaneous core and coating failure, while IPS e.max veneers show complete delamination between the two layers, before failure. The other specimens show mixed failure mechanisms.

**Figure 2:**
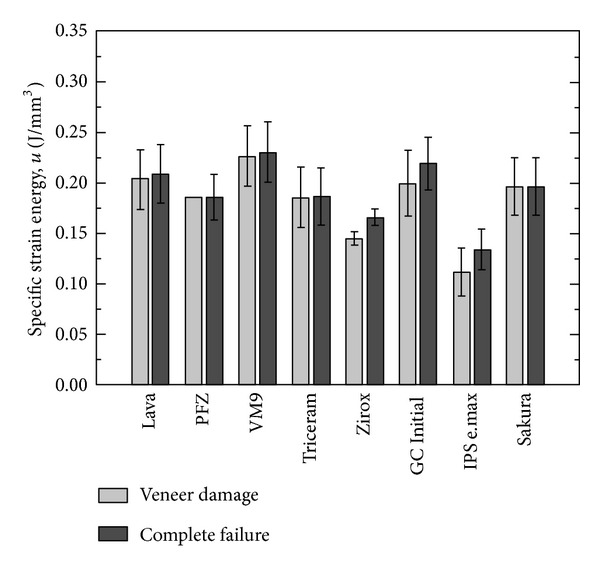
Strain energy per unit volume at failure and veneer damage for the two-layered specimens.

In Figure 2, the strain energies per unit volume of the two-layered specimens, which have been obtained from the experimentally measured force-deflection curves, are compared. In particular, Figure 2 shows the total strain energy at failure (*u*_tot*f*_), that is, when *P* = *P*_*f*_, as well as the strain energy at specimen damage *u*_tot*p*_, that is, for *P* = *P*_*p*_. It is worth noting that the first peak load is considered to calculate *u*_tot*p*_ when the load displacement curve has multiple peaks, that is, in the case of serrated fracture (Figure 2).

Figure 2 shows that Vita VM9 ceramics provide the best mechanical behavior; it also shows that Ceramco PFZ and Sakura Interaction veneers have the same values for the two energies, *u*_tot*f*_ and *u*_tot*p*_, as a consequence of simultaneous failure of zirconia and veneer. Finally, IPS e.max, GC Initial ZR, and Zirox veneers show marked differences between *u*_tot*f*_ and *u*_tot*p*_ as a consequence of an early damage of the ceramic coating, due to complete delamination or fracture.

## 4. Discussion

The zirconia-based ceramic materials are the most promising for dental application, because of their transformation toughening capabilities [5, 6]. In particular, this action can be mainly ascribed to a stress-induced tetragonal-to-monoclinic phase transformation (*t* → *m*) and to the corresponding volume expansion [7]. More specifically, the latter induces compressive internal stresses, thereby leading to crack growth arrest and to an increase in fracture toughness [8, 9]. It is worth noting that zirconia-based ceramics are usually doped with stabilizing oxides, such as yttrium (Y-TZP, Yttria Tetragonal Zirconia Polycrystal), in order to ensure a tetragonal structure at room temperature and, therefore, improve this toughening effect. As a direct consequence of these interesting features, many research activities have been carried out on this subject so far, with the aim of analyzing the mechanical strength of Y-TZP dental ceramics, as well as their interaction with different ceramic veneers. In detail, due to the brittle behavior of this class of materials, several experiments have been conducted in order to understand the effects of different surface conditions on their mechanical strength [10–13]. Furthermore, as Y-TZP ceramics are used in combination with veneering ceramics to produce all-ceramic restorative systems, the mechanical behavior of two-layered structures has also been recently investigated by suitable experimental tests [14–17].

In the present study, the authors analyzed the mechanical behavior of a commercial yttria-stabilized zirconia for dental application (Kavo Everest Bio ZS Blank), as well as its interaction with eight commercial veneering ceramics.

The effects of surface roughness as well as of the coloring process on the mechanical strength of the stabilized zirconia, analyzed by standard three-point bending tests, were firstly examined. The results indicate that surface roughness plays a critical role in the mechanical strength of zirconia structures, because a strong increase in the average flexural strength, from about 700 MPa to 1000 MPa, is observed after a mechanical polishing treatment of the test specimens. The results also show that the flexural strength of the polished specimens is characterized by smaller standard deviations (and higher *Weibull* moduli). More accurate surface polishing, that is, smaller values of surface roughness, could further increase the mechanical strength of the material [10–12]; however, this has a limited practical implication due to the higher values of surface roughness produced by the CAM milling processes, as well as by the common postmilling laboratory procedures [10–12]. 

Furthermore, the results show that the coloring process, carried out by using a commercial coloring liquid (*Zirkon Zahn*), has no significant effects on the mechanical strength of the zirconia specimens. As zirconia-based structures are normally used in combination with veneering ceramics for the production of all-ceramic restorations, the mechanical behavior of eight commercial ceramic veneers has been analyzed by three-point bending tests of two-layered zirconia-veneer specimens, and comparative studies of the selected ceramics have been carried out. The results achieved by means of bending tests show the following three different failure mechanisms for the selected ceramic veneers [5–15]: (i) simultaneous failure of zirconia and veneer, indicating a good adhesion strength between core and veneer; (ii) complete interfacial delamination, indicating a lower adhesion strength; (iii) serrated fracture, characterized by mixed cohesive and adhesive failure mechanisms. Furthermore, the total strain energy per unit volume of the two-layered specimens was calculated to give an overall measure of the mechanical behavior of the eight selected core/veneer systems.

It is worth noting that the effects of surface roughness, at the core/veneer interface, on the adhesion mechanisms were not analyzed in this investigation; however, even if it is expected to play a significant role in failure mechanisms of all-ceramic restorations, the surface morphology of the zirconia dental frames, obtained from CAD-CAM techniques, is not modified prior to ceramic veneering, due to both economic and technological issues. Therefore, this work was aimed at the identification of the best combinations of core/veneer, based on a reference surface condition, with roughness values (*R*_*a*_) close to the that obtained from CAM milling process [11].

On the contrary, the gingival surfaces of dental bridges are normally not veneered and, consequently, the roughness of the zirconia frame plays a very important role in the mechanical strength, because they are subjected to tensile stresses during chewing; therefore, high care should be devoted to both milling and/or possible postmilling laboratory procedures of zirconia frames in order to avoid the formation of rough surfaces, especially in the connector area, where geometric discontinuities and sources of stress concentration are present.

## 5. Conclusions

The results of this research indicate that close attention should be paid to the preparation procedure of zirconia-based crowns and bridges by CAD/CAM process, with the aim of obtaining smooth surfaces, because a strong effect of surface roughness on the mechanical strength was observed. Furthermore, no significant effects of the coloring process on the mechanical behavior were measured. Finally, comparative studies of several commercial ceramic veneers, to be used for the realization of all-ceramic systems, show different mechanical behavior and failure modes between the selected veneers. Further studies should be carried out to measure the wear properties of the ceramic veneers, as well as the interaction with natural enamel.

## Figures and Tables

**Table 1:** Nomenclature of zirconia core specimens.

Specimen type	Description
Type A	Sintered
Type B	Colored and sintered
Type C	Sintered and polished
Type D	Colored, sintered, and polished

**Table 2:** Flexural strength and surface roughness for the four types of zirconia specimens.

Specimen type	Surface condition, Ra (*μ*m)	Flexural Strength (MPa)		Weibull parameters (MPa)	
		Average	SD	Modulus	Characteristic strength
Type A	1.75 ± 0.47	688	100	8	729
Type B	1.27 ± 0.36	733	109	7	779
Type C	0.13 ± 0.03	982	75	15	1005
Type D	0.12 ± 0.03	991	46	22	1007

**Table 3:** Failure mechanism of two-layered zirconia-veneer specimens.

Veneer type	Failure mechanism*
Lava Ceram	*F* and *S *
Ceramco PFZ	*F *
Vita VM9	*F* and *S *
Triceram	*F* and *S *
Zirox	*S* and *D *
GC Initial ZR	*F* and *S *
IPS e.max	*D *
Sakura Interaction	*F *

*F: simultaneous failure, *S*: serrated fracture, and *D*: complete delamination.
